# A Retrospective Study on Pain Sensitivity in Pediatric Patients with Severe Obstructive Sleep Apnea Undergoing Adenotonsillectomy

**DOI:** 10.5812/aapm-163436

**Published:** 2025-08-17

**Authors:** Naila Ahmad, Daniel Roke, Jill Kilkelly, Tatyana Demidovich, Andrea Zepeda, Andrew Oster, Marion F Svendrowski, Pin Yue

**Affiliations:** 1 School of Medicine, Saint Louis University, St. Louis, Missouri, USA; 2 SSM Health Cardinal Glennon Medical Center, St. Louis, Missouri, USA; 3 SSM Health Saint Louis University Hospital, St. Louis, Missouri, USA

**Keywords:** Obstructive Sleep Apnea, Pain Sensitivity, Adenotonsillectomy, Sex Differences, Pediatric

## Abstract

**Background:**

Regarding post-adenotonsillectomy pain management, pediatric patients with severe obstructive sleep apnea (OSA) are often treated with lower total opioid doses, and with more short-acting opioid types, relative to their non-severe OSA counterparts. It is unclear whether this practice undermines these patients’ pain management and exposes them to a traumatic experience due to under-managed surgical pain. Pain sensitivity differs between adult males and females. Whether the difference exists in the pediatric population, especially related to surgical pain sensitivity, is unclear.

**Objectives:**

We explored the differences in pain sensitivity between sexes using a pediatric adenotonsillectomy population and studied the effects of sleep apnea on acute pain responses.

**Methods:**

We retrospectively analyzed perioperative pain management approaches in a pediatric adenotonsillectomy surgical population at our medical center. Patients aged 3 to 12 years were grouped into those with less severe OSA or severe OSA. Perioperative pain medications and post-anesthesia care unit (PACU) pain assessments were compared between two groups.

**Results:**

Of a total 3,586 patients we analyzed, a higher percentage of severe OSA patients received non-opioid type analgesic medications, as well as fewer and shorter-acting opioids. The use of rescue opioids in the post-operative period did not significantly increase in patients with severe OSA. Post-operative pain scores did not differ between patients with and without severe OSA status. Female patients had significantly higher post-operative pain scores than males in the less severe OSA group, but sex differences in the severe OSA group were not significant.

**Conclusions:**

Despite receiving less perioperative opioid, pediatric patients with severe OSA did not demonstrate worse pain scores in post-surgical recovery. Overall, we did not under-manage their post-operative pain with reduced dosages of opioids. We did find that a sex difference in acute pain perception exists even in pre-puberty pediatric population. However, severe OSA reduces pain sensitivity in both sexes, and blunts sex differences in acute pain perception.

## 1. Background

Obstructive sleep apnea (OSA) is defined as a syndrome with periodic, partial, or complete obstruction of the upper airway, causing disruption of normal sleep patterns, intermittent hypoxia, episodic hypercarbia, pulmonary and cardiovascular dysfunction (1). Studies have demonstrated a strong link between OSA and incidental cardiovascular diseases such as hypertension, myocardial infarction (MI), stroke, and all-cause mortality (1, 2). The estimated prevalence of moderate to severe OSA [Apnea-hypopnea Index (AHI) > 10] is about 10% in adult men, 9% in adult women, and 1 - 4% in the pediatric population (1-3), yet the numbers may be underestimated because of underdiagnosis and increasing prevalence of obesity (4). 

Surgical intervention has been a common practice to treat OSA and sleep-disordered breathing in the pediatric population (2, 5, 6). However, adenotonsillectomy is associated with significant post-operative pain, as well as nausea and vomiting (7). Severe OSA is also associated with increased sensitivity to opioids, with clear implications for post-tonsillectomy respiratory depression and pain management (6, 8). Due to these concerns, patients with moderate to severe OSA tend to receive less perioperative opioid for pain management (6). However, inadequate post-operative pain management causes distress, fear, anxiety, and behavioral issues. These physical and psychological responses to pain not only affect children's health directly but may also predispose them to developing chronic pain in the future (9-11). Therefore, good quality acute post-operative pain management has paramount importance in adenotonsillectomy patients. To date, there is no systemic evidence to show whether we provide adequate analgesia in pediatric patients with severe OSA. 

Meanwhile, there is well-documented difference in pain sensitivity between adult males and females. In general, females are more sensitive to pain stimulation from external sources such as pressure or temperature (12-14). Yet, it is unclear whether the same trend holds true for acute post-surgical pain, especially in pediatric populations. Severe sleep apnea enhances pain sensitivity via activating inflammatory response pathways but also precipitates a higher sensitivity to opioid analgesic potency (15, 16). It is unclear whether the difference in acute pain perception differs between sexes with severe obstructive sleep disorders. 

## 2. Objectives

In this retrospective analysis, we sought to discover answers to these questions by investigating whether (1) we undertreat post-operative pain in pediatric patients with severe OSA, or (2) response to surgical pain stimulation in severe OSA patients differs between males and females.

## 3. Methods

### 3.1. Experimental Design and Analysis

This is a retrospective cohort study. The study protocol was approved by the Saint Louis University Institutional Review Board (IRB#27602) and SSM Health Research Business Review Board as a clinical quality improvement project. Data was retrieved from the Electronic Health Records (EPIC System, Verona, WI) with all personal identification information removed by the SSM IT team following proper HIPAA guidance.

### 3.2. Study Population

Pediatric patients including in this study were children aged 3 - 12 years who underwent elective adenotonsillectomy at Cardinal Glennon Children's Medical Center in St. Louis, Missouri from July 2012 to June 2017. For this period, we implemented a standard anesthesia protocol which offered patients few intra- and post-operative pain management options. Opioid regimens included short acting fentanyl and longer acting morphine, as dexmedetomidine and other, newer non-opioid analgesics had not yet been introduced into pediatric clinical practice. Exclusion criteria included those patients with incomplete medical records, and unplanned admission due to adverse events other than uncontrolled pain. 

### 3.3. Obstructive Sleep Apnea Classification and Anesthesia Management

Patients arrived on the day of surgery to undergo elective adenotonsillectomy under general anesthesia. These patients were either diagnosed with OSA from standard overnight polysomnography (PSG) or had sleep-disordered breathing or chronic tonsillitis. Per hospital policy, patients were to be discharged home if they had no PSG or mild OSA (AHI < 10) (2) unless they had post-operative complications. If patients had severe OSA (AHI > 10), they were admitted overnight in the hospital for post-anesthesia observation with continuous Pulse Oximetry monitoring. Therefore, we classified the patient population as having severe OSA (AHI > 10) or less-severe OSA based on their post-operative admission status. The less-severe OSA group included patients who were discharged the same day after adenotonsillectomy, and severe OSA group consisted of patients who stayed overnight due to their AHI status (AHI > 10) or post-operative respiratory complications. However, some patients in the less-severe OSA group might have had undiagnosed severe OSA since they did not have PSG study. 

In the preoperative area, some patients were given per os acetaminophen at 10 - 15 mg/kg or midazolam at 0.3 - 0.5 mg/kg up to 15 mg per anesthesiologist’s assessment. General anesthesia included inhalation induction with 70% N_2_O with sevoflurane, peripheral IV access, intubation, and was maintained with sevoflurane with 30 - 50% oxygen. Intra-operatively, the patient received ~10ml/kg IV fluid, opioids for pain, and dexamethasone and ondansetron for post-operative nausea and vomiting (PONV). At the end of the case, the patient was extubated awake, transferred to the post-anesthesia care unit (PACU) for observation. In the PACU, patients were assessed for pain level using either the FLACC Scale or the Wong-Baker FACES Scale if they were under 7 years old (17, 18). If the patient was over 7 years, the Visual Analogue Scale (VAS) was used (19). Patients were also assessed for their physical status of recovery using the Aldrete Score system (20). A patient was considered discharge/step down ready when they had an Aldrete score above 7. These assessments were done every 15 minutes until the patient was discharged or transferred to the floor for further observation. The attending anesthesiologists would prescribe more pain medications based on their discretion in the PACU. 

### 3.4. Data Analysis

 Data analysis was conducted using SAS (Version 9.4, SAS Institute, Cary, NC). In this study, most patients received either fentanyl citrate (F) or morphine sulfate (M) as intra- and post-operative opioids. Sixteen patients also received hydromorphone HCl (H) as post-operative pain medicine. Opioid equivalence-analgesia has been studied by multiple groups with some variations (21, 22). For simplicity, we used morphine equivalent conversion published by Wen et al., calculated as F:M:H at 0.135:10:2 ratio, and expressed as mg/kg Equivalent (mg/kg Eqv) (21). 

The continuous variables were described according to their statistical distribution by mean ± standard deviation. Normality was tested using the Shapiro-Wilk test. Comparisons between groups (less-severe OSA and severe OSA) were done using (1) for quantitative variables: The analysis of variance (ANOVA) test, or the Kruskal-Wallis test when assumptions for the ANOVA test were not met; (2) for categorical parameters: Chi-squared test.

## 4. Results

A total of 3,586 patients met our inclusion criteria, and 481 patients were classified as having "severe OSA" since they were admitted for overnight observation according to protocol. Demographic characteristics of the two groups were listed in Table 1. There were no differences in age and gender distribution between the two groups. Patients with severe OSA had a slightly longer OR time (P = 0.0613), but PACU recovery time was comparable between the two groups. The severe OSA group had significantly higher American Society of Anesthesiologists (ASA) scores and BMI.

**Table 1.** Demographic Characteristics of the Two Cohorts

Variables	Less-Severe OSA (N = 3105)	Severe OSA (N = 481)	P-Value
**Age (y)**	6.5 ± 2.8 ^a^	6.6 ± 2.5	0.3341
**Gender (F/M)**	1618/1487	237/244	0.2466
**BMI (kg/m** ^**2**^ **)**	18.5 ± 4.7	20.9 ± 7.4	< 0.0001
**ASA Scores**	1.9 ± 0.4	2.3 ± 0.5	< 0.0001
**OR time (min)**	47.9 ± 20.6	54.4 ± 27.7	0.0613
**PACU time (min)**	88.9 ± 37.0	84.2 ± 44.7	0.2547

Abbreviations: OSA, obstructive sleep apnea; ASA, American Society of Anesthesiologists; PACU, post-anesthesia care unit.

^a^ Values are expressed as mean ± standard deviation (SD).

### 4.1. Reduced Intra-operative and Post-operative Opioid Use in Patients with Severe Obstructive Sleep Apnea

Our data showed that multimodal analgesia was more commonly used in patients with severe OSA. A higher percentage of severe OSA patients received preoperative acetaminophen, but a lower percentage were given oral midazolam (Table 2). For intra-operative pain management, patients with severe OSA received less opioid overall, and tended to receive short-acting opioid types. Our results showed that a higher percentage of patients were administered fentanyl instead of morphine intra-operatively (Table 2) with reduced dosage (morphine equivalence 0.06 mg/kg Eqv vs 0.1 mg/kg Eqv, P < 0.001). Patients with severe OSA received less postoperative opioid also (Table 2 postoperative morphine equivalence 0.061 mg/kg Eqv vs 0.067 mg/kg Eqv). About 5% of patients with severe OSA did not receive any narcotics, while all patients in the "Less-severe OSA" group received at least one form of opioid (Table 2). 

**Table 2.** Pain Management Related Medicines and Dosing Characteristics ^a^

Variables	Less-Severe OSA (N = 3105)	Severe OSA (N = 481)	P-Value
**Pre-op acetaminophen**	67.9	83.9	< 0.001
**Pre-op midazolam**	39.6	28.9	< 0.001
**Intra-op fentanyl usage**	13.1	35.9	< 0.001
**Intra-op morphine usage**	49.7	31.5	< 0.001
**Intra-op morphine/fentanyl usage**	37.2	27.8 ^b^	0.0105
**Intra-op morphine equivalence (mg/kg)**	0.102 ± 0.042	0.061 ± 0.047	< 0.001
**Post-op morphine equivalence (mg/kg)**	0.067 ± 0.041	0.061 ± 0.044	0.0095

Abbreviation: OSA, obstructive sleep apnea.

^a^ Values are expressed as percentage or mean ± standard deviation (SD).

^b^ 4.8% of individuals with severe OSA did not receive any narcotics.

### 4.2. Lower or Equivalent Post-anesthesia Care Unit Pain Scores in Patients with Severe Obstructive Sleep Apnea

Despite our findings that severe OSA patients received less total opioid intra-operatively, and tended to receive only short-acting opioid, these patients did not show significant increases in postoperative pain scores (Table 3). In general, severe OSA patients did not receive more rescue narcotic during their PACU stay; their postoperative morphine equivalences were less compared to less severe OSA patients (Table 2 post-op morphine 0.061 mg/kg Eqv vs 0.067 mg/kg Eqv, P = 0.0095). When 15-min, 30-min, 60-min, and max PACU pain scores were compared between the two groups, there were no significant differences (Table 3). Patients with severe OSA had significantly lower post-PACU (step down unit) pain scores. However, severe OSA patients had significantly lower PACU-discharge scores (Aldrete score), suggesting they had slow recovery from the anesthesia event (Table 3). 

**Table 3.** Comparison of Pain Scores and Other Post-anesthesia Care Unit Characteristics Between Severe Obstructive Sleep Apnea and Less-Severe Obstructive Sleep Apnea Groups ^a^

Variables	Less-Severe OSA	Severe OSA	P-Value
**15-min PACU pain score**	3.5 ± 3.0	3.5 ± 2.8	0.6144
**30-min PACU pain score**	3.3 ± 2.7	3.3 ± 2.5	0.9061
**60-min PACU pain score**	2.1 ± 2.0	1.9 ± 2.1	0.1094
**Max pain score in PACU**	4.7 ± 2.6	4.9 ± 2.2	0.3376
**Max pain score post PACU**	1.1 ± 1.7	0.1 ± 0.7	< 0.001
**First PACU Aldrete score**	6.6 ± 1.2	6.6 ± 1.2	0.6462
**PACU discharge Aldrete score**	8.0 ± 0.2	7.8 ± 0.3	< 0.001

Abbreviations: OSA, obstructive sleep apnea; PACU, post anesthesia care unit.

^a^ Values are expressed as mean ± standard deviation (SD).

### 4.3. Sex Differences in Opioid Usage, Post-operative Pain Scores

When the dataset was separated by sex, female patients were older with higher BMI (P < 0.001) but had comparable ASA scores (P = 0.036). Male and female patients did not differ in intra- and post-operative opioid usages (intra- and post-operative opioid equivalent, Table 4). 

**Table 4.** Sex Differences in Intra-operative and Post-operative Pain Assessment ^a^

Variables	Overall			Less-Severe OSA			Severe OSA		
	Male (N = 1731)	Female (N = 1855)	P-Value	Male (N = 1487)	Female (N = 1618)	P-Value	Male (N = 244)	Female (N = 237)	P-Value
**Age (y)**	6.3 ± 2.5	6.8 ± 2.6	< 0.001	6.3 ± 2.4	6.8 ± 2.5	< 0.001	6.1 ± 2.8	6.8 ± 2.8	0.003
**BMI (kg/m** ^**2**^ **)**	18.4 ± 4.8	19.1 ± 5.4	< 0.001	18.1 ± 4.5	18.7 ± 4.8	< 0.001	19.9 ± 6.8	21/9 ± 7.9	0.004
**ASA score**	1.99 ± 0.41	1.95 ± 0.41	0.036	1.9 ± 0.4	1.9 ± 0.4	0.101	2.3 ± 0.5	2.3 ± 0.5	0.170
**Intra-op opioid equivalence (mg/kg) ** ^**b**^	0.10 ± 0.96	0.09 ± 0.79	0.849	0.11 ± 1.06	0.10 ± 0.84	0.853	0.06 ± 0.04	0.06 ± 0.05	0.774
**Post-op opioid equivalence (mg/kg)**	0.07 ± 0.043	0.07 ± 0.04	0.089	0.07 ± 0.04	0.07 ± 0.04	0.389	0.07 ± 0.05	0.05 ± 0.04	0.021
**15-min PACU pain score**	3.4 ± 2.9	3.6 ± 2.9	0.011	3.4 ± 2.9	3.7 ± 3.0	0.017	3.3 ± 2.6	3.5 ± 2.9	0.364
**30-min PACU pain score**	3.0 ± 2.6	3.5 ± 2.7	< 0.001	3.0 ± 2.6	3.5 ± 2.7	< 0.001	3.1 ± 2.4	3.3 ± 2.6	0.258
**60-min PACU pain score**	2.0 ± 2.0	2.2 ± 2.1	0.009	2.0 ± 2.0	2.2 ± 2.1	0.084	1.7 ± 1.9	2.2 ± 2.2	0.049
**Max pain score in PACU**	4.6 ± 2.5	4.9 ± 2.6	< 0.001	4.6 ± 2.0	4.9 ± 2.6	< 0.001	4.8 ± 2.0	4.9 ± 2.3	0.785
**First PACU Aldrete score**	6.5 ± 1.2	6.6 ± 1.2	0.052	6.5 ± 1.2	6.6 ± 1.2	0.128	6.5 ± 1.3	6.7 ± 1.2	0.158
**PACU discharge Aldrete score**	8.0 ± 0.2	8.0 ± 0.2	0.620	8.0 ± 0.2	8.0 ± 0.2	0.900	7.9 ± 0.3	7.9 ± 0.3	0.450

Abbreviations: OSA, obstructive sleep apnea; ASA, American Society of Anesthesiologists; PACU, post-anesthesia care unit.

^a^ Values are expressed as mean ± standard deviation (SD).

^b^ Intra-op opioid equivalence (mg/kg): Fentanyl, morphine, and hydromorphone (H) were converted to opioid equivalent (mg/kg) according to the intra-operative converting formular described in methods section.

**Figure 1.**
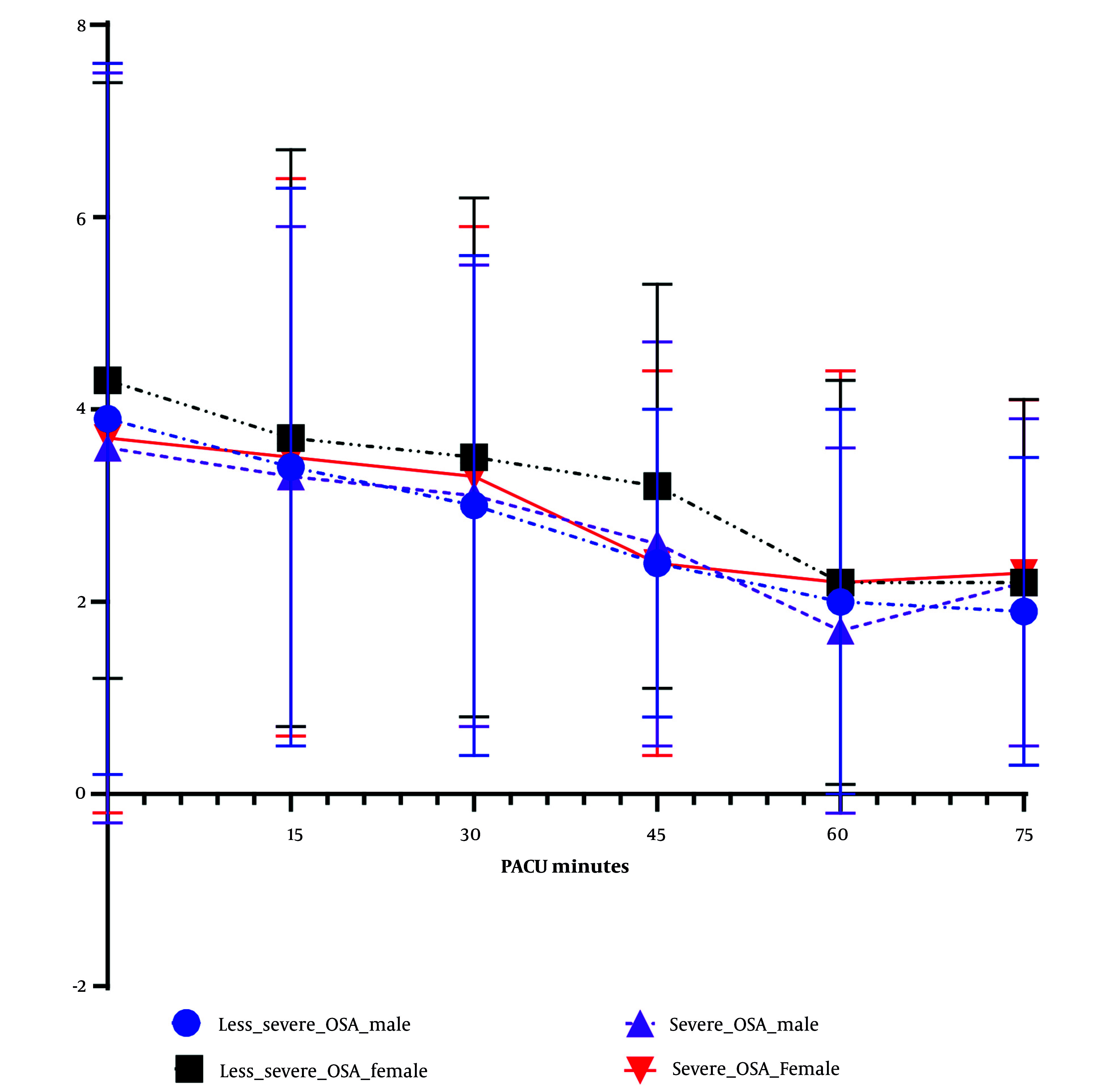
Post-anesthesia care unit (PACU) pain score over time among four groups of patients: Postoperative pain scores (FLACC score) were documented every 15 minutes after patient’s arrival. Average pain scores (± SD) were plotted over time among four groups: Male and female patients who were classified either with less-severe obstructive sleep apnea (OSA) or severe OSA. Overall, female patients demonstrated higher PACU pain scores, but FLACC scores between female and male in severe OSA group did not statistically differ crossing PACU stay.

Overall, female patients had significantly higher post-operative pain scores (Table 4 and Figure 1). When postoperative pain scores were grouped by severity of OSA, female patients had significantly higher postoperative pain scores than males in the less-severe OSA group, but sex difference in postoperative pain diminished the severe OSA group (Table 4 and Figure 1). Between male and female patients, there was no significant difference of final score to be discharged (Aldrete score, P = 0.620, Table 4). 

## 5. Discussion

To answer the questions as to whether (1) we undermanaged post-tonsillectomy pain in children with severe OSA and (2) the response to surgical pain stimulation in severe OSA patients differs between males and females, we retrospectively analyzed clinical data from pediatric patients who had elective adenotonsillectomy in the institute. We found that children with severe OSA tend to receive less opioid overall, with an emphasis on use of short-acting opioids, for their post-operative pain management. However, these practices did not affect their postsurgical pain: These patients had similar discharge time, PACU pain scores, and use less rescue pain medications (Tables 2 and 3). While female patients in less-severe OSA group demonstrated higher acute pain scores in PACU than males, the sex differences in PACU pain scores were similar in the severe OSA group. 

Post-adenotonsillectomy pain management is a major challenge in the goal to improve quality of care in children (7, 23). Poorly controlled PACU pain is associated with agitation, delayed discharge, unplanned hospital admission, low patient satisfaction, and even long-term psychological issues (7). The importance of proper pain management is profound. However, in patients with severe OSA, airway obstruction disrupts the regular diurnal sequence, causing chronic intermittent hypoxia, inducing oxidative stress and the production of reactive oxygen species (15, 16). Clinically, OSA contributes to hyperalgesia as well as a greater response to administration of exogenous opioids, such as significant opioid-induced respiratory suppression (1, 6, 8). Due to these concerns, adenotonsillectomy patients with moderate to severe OSA tend to receive less perioperative opioid for pain management. Using short-acting opioids such as fentanyl is also a common practice (7, 15). The ASA encourages adjusting opioid dosage in OSA patients. Our data demonstrates that even though we used less opioid in children with severe OSA, we did not undermanage their PACU pain. Neither discharge pain score nor maximum PACU pain score of OSA patients showed significant differences from the mild and undiagnosed groups (Table 3). Indeed, the discharge readiness indicator Aldrete score was lower for the severe OSA group, suggesting these patients might be less awake from general anesthesia or from opioid usage. 

It is unclear whether pediatric patients with severe OSA have higher pain tolerance or are more sensitive to opioids. Brackley et al. using a rat model demonstrated that intermittent hypoxia induced elevated endogenous opioids levels, which results in a “relative” overdose of exogenous opioids (24). Brown et al. suggested that pediatric OSA patients require less opioid due to upregulation of opioid receptors (8). These studies suggest that OSA patients tend to be sensitive to opioid administration. However, 4.8% of patients with severe OSA did not receive any opioid intra- and post-tonsillectomy procedure without exacerbating surgical pain, suggesting they have higher pain tolerance or impaired pain sensitivity. Overall, our data supports the approach of utilizing less opioid to manage acute surgical pain in patients with a history of severe OSA. 

Differences in pain perception between adult males and females have been observed by several clinical studies (25, 26). Several factors, including sex hormones, sex-related cortical differences between male and female, genetic composition, and psychosocial mechanisms, could be in play in the pain sensitivity differences (26). However, studies suggest that clinical pain conditions show no difference in prevalence in children before reaching puberty (23). Our analysis shows female pediatric patients have higher post-operative pain scores without increased opioid consumption, suggesting sex difference in acute pain perception exists before puberty in general. However, severe OSA blunts sex difference of pain sensitivity in this population. In patients with severe OSA, post-operative pain scores are not significantly different between male and female. It is unclear what the biological mechanism of this alteration is. 

Patients with severe OSA have hyperalgesia because of fragmented sleep and hypoxemia that enhance sensitivity to pain, promote inflammation, and increase spontaneous pain (2, 5, 16). Meanwhile, these patients are highly sensitive to opioid administration (6). There is limited literature investigating sex differences in acute pain sensitivity in children, especially in children with sleep apnea symptoms. We are unsure about factors that could contribute to the difference in acute pain between sexes in the pediatric population. However, the combination of higher opioid sensitivity/higher pain tolerance could be the contributing factors that decrease sex difference in pain sensation when severe OSA is a consideration. Further studies are needed to investigate this topic and may provide further clinical guidelines for acute pain management in the pediatric population. 

There were several limitations in this study. First, as a retrospective cohort study, we cannot evaluate the degree of bias of the selection cohorts. First, the group of OSA classification was arbitrary; many patients without sleep studies could potentially have severe OSA but be misclassified due to lack of proper sleep studies. Second, the records of post-operative pain scores and Aldrete scores were done by PACU nurses with varying degrees of training, therefore potentially lacking consistency. However, we had a relatively large sample size, and we carefully selected a time period during which we had relatively uniform anesthesia practice. We thereby decreased confounding factors such as new multimodal approaches using agents like dexmedetomidine and ketorolac, that have now become common components of acute postsurgical pain management in adenotonsillectomy patients. 

Overall, our study supports the idea that we did not undertreat OSA patients for their post-operative pain because of their higher analgesic sensitivity to opioids. We also found that sex difference in acute pain sensation exists in pediatric population. However, severe OSA can blunt difference in acute pain perception between female and male children.

## Data Availability

The dataset presented in the study is available on request from the corresponding author during submission or after publication. The data are not publicly available due to intellectual property owned by the SSM Health.
